# In this month’s Bulletin

**DOI:** 10.2471/BLT.17.000117

**Published:** 2017-01-01

Margaret Chan, Erik Solheim and Petteri Taalas (2) explain how the World Health Organization, the United Nations Environment Programme and the World Meteorological Organization work together on the environmental causes of ill health.

Ishani Pathmanathan et al (3) explain why screening and treatment for tuberculosis is an integral part of care for people living with HIV.

Jan Dirk Herbermann and Fiona Fleck (6–7) report on efforts to prevent both targeted and inadvertent destruction of health-care capacity in conflict settings. Peter Salama tells Fiona Fleck (8–9) how the World Health Organization’s new emergencies programme is designed to help countries respond to crises. 

## Global

### Preventing interpersonal violence

Christopher R Mikton et al. (36–48) propose research priorities.

### Travel restrictions during the 2013–2016 Ebola outbreak

Wendy Rhymer and Rick Speare (10–17) evaluate countries’ response to WHO’s travel recommendations.

### Biological outcomes and behavioural risks 

Maureen Miller and Emily Hagan (62–68) describe other aspects of surveillance needed for pandemic prevention.

### Bombing the hospital

Preeti Patel et al. (79–81) discuss motives for – and prevention of – attacks on healthcare facilities and staff.

### Not settling for less

Bejoy Nambiar et al. (76–78) explain why quality improvement is an essential part of health-care delivery.

## China

### Planning for an ageing population

Junfang Xu et al. (18–26) estimate the cost of illness attributable to dementia. 

## Belgium

### Finding rare cases

G Suzanne A Smit et al. (27–35) measure costs and effectiveness of tuberculosis screening programmes.

## Bangladesh, Brazil, India, Nepal, Pakistan, Peru, South Africa and the United Republic of Tanzania

### Quantifying inappropriate use

Elizabeth T Rogawski et al. (49–61) measure episodes of antibiotic treatment in children. 

## Pacific Island countries and territories 

### The limits of surveillance 

Adam T Craig et al. (69–75) analyse transmission of Zika virus from 2007 to February 2015. 

